# Understanding the factors shaping Healthy Life Expectancy (HALE) in Indigenous Australians: A Scoping Review

**DOI:** 10.1371/journal.pone.0347718

**Published:** 2026-11-11

**Authors:** Md Mashiur Rahman, Md. Nuruzzaman Khan, Mahmudul Hassan Al Imam, Shimlin Jahan Khanam, Gulam Khandaker, Delwar Akbar

**Affiliations:** 1 School of Business and Law, Central Queensland University, Australia; 2 Department of Population Science, Jatiya Kabi Kazi Nazrul Islam University, Mymensingh, Bangladesh; 3 Nossal Institute for Global Health, Melbourne School of Population and Global Health, The University of Melbourne, Australia; 4 Central Queensland Public Health Unit, Central Queensland Hospital and Health Service, Australia; 5 School of Health, Medical and Applied Sciences, Central Queensland University, Australia; 6 National Centre for Immunisation Research and Surveillance, Sydney, Australia; 7 Discipline of Child and Adolescent Health, Sydney Medical School, The University of Sydney, Australia; Ulster University - Coleraine Campus: Ulster University, UNITED KINGDOM OF GREAT BRITAIN AND NORTHERN IRELAND

## Abstract

**Background:**

Aboriginal and Torres Strait Islander peoples in Australia experience substantial inequities in life expectancy (LE) and Healthy Life Expectancy (HALE) compared with non-Indigenous Australians. These disparities are shaped by complex, interrelated physical, mental, socio-economic, and regional determinants across the life course. This scoping review aims to examine these factors and their influence on HALE among Indigenous Australians, identifying key patterns, gaps, and implications for public health policy and research.

**Methods:**

A scoping review was conducted following the PRISMA-ScR framework. We systematically searched seven academic databases (MEDLINE, Scopus, Web of Science, ScienceDirect, PsycINFO, CINAHL and the Social Science). We also searched relevant grey literature sources published between January 2015 and April 2025. The inclusion criteria focused on Aboriginal and Torres Strait Islander peoples in Australia and reported HALE, LE, physical, mental, socioeconomic or regional factors relevant to years lived in good health. Data were charted using a standardized extraction framework to capture study characteristics and key findings across these domains.

**Results:**

Twenty-two studies were (11 peer-reviewed studies and 11 government reports) included in this review. Evidence consistently showed that chronic disease burden, multimorbidity, and limited access to preventive care were associated with reduced HALE among Aboriginal and Torres Strait Islander peoples. Mental health conditions, including psychological distress and depression, contributed substantially to years lived in poor health and influenced health behaviours and service engagement. Socio-economic disadvantage particularly in remote communities with constrained health system capacity, further compounded HALE disparities among Aboriginal and Torres Strait Islander peoples.

**Conclusion:**

The review identified physical, mental, socio-economic, and regional conditions relevant to HALE among Aboriginal and Torres Strait Islander peoples. Nationally comparable HALE estimates, disaggregated by age and region, are needed to identify and monitor inequalities in years lived in good health.

## Introduction

Aboriginal and Torres Strait Islander peoples in Australia experience persistent health disparities compared to non-Indigenous populations [1]. These disparities are reflected in significant gaps, relative to non-Indigenous Australians in both life expectancy (LE, estimated lifespan a newborn baby is likely to achieve, assuming birthtime mortality rate remains constant) and Healthy Life Expectancy (HALE, number of years an individual is expected to live in full health, excluding years lived in poor health across any dimension) [2–4]. Such disparities are rooted in a long history of colonisation, dispossession, forced removals, and cultural suppression [5,6]. They are further compounded by intergenerational trauma and reduced trust in health institutions [5,7]. Compared with non-Indigenous Australians, Aboriginal and Torres Strait Islander peoples experience higher morbidity and disability, limited access to healthcare, and more years lived in poor health [7]. Importantly, these inequalities persist despite national initiatives aimed at closing the gap in health outcomes, including improvements in years of life lost (YLL), years lived with disability (YLD), and disability-adjusted life years (DALYs) [8].

Existing Australian literature identifies a wide range of factors that may contribute to disparities in LE and HALE among Aboriginal and Torres Strait Islander peoples. These factors extend across multiple domains, including physical health, mental health, and social disadvantages. Physical health issues are marked by disproportionately high rates of cardiovascular disease, diabetes, chronic kidney disease, and respiratory illnesses, with earlier onset compared to non-Indigenous people [7,9,10]. Mental health challenges particularly depression, psychological distress, and intergenerational trauma are also prevalent and remain insufficiently addressed, especially among Indigenous elders [11,12]. These physical and mental health burdens are compounded by socio-economic disadvantages, including lower income, limited educational attainment, poor housing conditions, and reduced employment opportunities [13,14]. Geographical locations further exacerbate inequalities: around 30% of First Nations people reported needing care but not accessing a provider, with more than half of those in remote areas citing service availability, transport difficulties, and distance as the primary barriers [8].

While these studies provide valuable insights into specific health dimensions, none addresses HALE as an integrated outcome across all four domains. Existing Australian reviews are consistently narrower in scope. Reviews of chronic disease prevention and management programs examine social and cultural determinants only in relation to program design and delivery, rather than HALE itself [15]. A review of racism and discrimination considers physical and mental health together but does not examine socio-economic or regional determinants, nor does it address HALE [16]. Quantitative decomposition studies of the HALE gap, including Zhao et al. [2], are limited to a single state and data source rather than synthesising evidence nationally across peer-reviewed research and government reports. Existing reviews and quantitative studies have generally focused on individual determinants, specific programs, or jurisdictions. Consequently, evidence relevant to HALE across physical, mental, socio-economic, and regional domains remains fragmented across peer-reviewed studies and government reports. This review brings these sources together to map the available evidence and identify important gaps. It addresses three questions: (1) What physical and mental health factors influence HALE? (2) How do socio-economic conditions shape HALE outcomes? and (3) How do regional disparities affect HALE among Aboriginal and Torres Strait Islander peoples?

## Methods

This scoping review was conducted in accordance with the Preferred Reporting Items for Systematic Reviews and Meta-Analyses extension for Scoping Reviews (PRISMA-ScR) [17]. The available literature addressing all dimensions of health, including physical, mental, and social well-being among Aboriginal and Torres Strait Islander peoples in Australia was included and systematically mapped, then critically summarised.

### Database sources

A comprehensive search of relevant literature was conducted in April 2025 across seven databases: MEDLINE, Scopus, Web of Science, ScienceDirect, PsycINFO, CINAHL, Social Science Database. The search was conducted using pre-developed strategies created in consultation with the authors and a specialised librarian. The search strategy included terms related to HALE and LE combined with population-specific terms (e.g., “Aboriginal,” “Torres Strait Islander,” “First Nations”), with the study context restricted to Australia (S1 Table). We did not specifically include any terms related to health and social aspects to ensure that all relevant papers were captured and that the terms used did not inadvertently exclude any studies. All searches were limited to English-language publications from January 2015 to April 2025. Boolean operators (AND, OR) were used to combine the terms. Additional searches were conducted in Google, Google Scholar, and relevant databases and the relevant Australian government websites, including Australian Institute of Health and Welfare [AIHW], Australian Bureau of Statistics [ABS], Productivity Commission [PC], Australian Government Department of Health and Aged Care, and the National Indigenous Australians Agency [NIAA]. Reference lists of the selected articles and reports were also thoroughly checked for additional papers.

### Study selection

All search results were imported into Covidence for automatic duplicate removal, followed by independent title, abstract and full-text screening by three reviewers (MR, MHAI and SJK). Discrepancies were resolved through discussion with the research team (DA, MHAI and NK).

### Eligibility criteria

Studies identified were reviewed based on the pre-defined inclusion and exclusion criteria (Table 1). The criteria were developed in line with the study’s objectives and agreed upon by all authors.

**Table 1:** Inclusion and exclusion criteria.

Criteria	Inclusion	Exclusion
Study Population	Aboriginal and Torres Strait Islander Australians;	non-Australian population-based study
Outcomes	Direct evidence comprised sources reporting HALE or LE estimates. Indirect evidence was eligible when it examined outcomes or determinants relevant to healthy years lived, including disease burden, disability, morbidity, quality of life, wellbeing, mental health, socioeconomic conditions or regional access to care.	Sources that reported neither HALE nor LE estimates nor evidence relevant to healthy years lived, including disease burden, disability, morbidity, quality of life, wellbeing, mental health, socio-economic conditions, or regional access to care.
Explanatory variables	Socio-economic determinants (education, employment, income, housing) and physical and mental health conditions influencing HALE and LE among Aboriginal and Torres Strait Islander peoples	Not included are socio-economic determinants, physical and mental health conditions
Study Type and design	Peer-reviewed articles, national reports, and government publications (e.g., ABS, AIHW). Relevant studies of any design were included.	Opinion pieces, editorials, and blog post were excluded.
Publication Date	Published from January 2015 to April 2025	Published before 2015
Language	English	Languages other than English

### Data extraction

After reviewing the included studies, we designed two tailored data extraction templates; one for peer-reviewed scientific articles and another for government reports. These separate templates allowed for comprehensive data capture, recognising that the two types of sources present information in different formats. Data extraction was conducted independently by two reviewers (MR and SJK), and disagreements were resolved through discussion with the research team. The data extraction templates were pilot tested on a subset of included studies to ensure consistency and completeness prior to full data charting. No additional data were sought from the study authors. Data items included study characteristics (author, year, study design, population, age group, sample size, and setting), outcome measures (HALE, LE, and quality of life), and key determinants across four domains: physical health, mental health, socio-economic factors, and regional characteristics. For synthesis, variables were grouped into these domains to enable comparison across heterogeneous sources.

### Data synthesis

Given the heterogeneity of study designs, data sources, and outcome measures across included scientific papers and government reports, a narrative synthesis approach was applied. Extracted data from Tables 2 and 3 provided contextual information to frame the evidence base, while S2 and S3 Tables informed the thematic analysis of physical, mental, and socio-economic factors influencing HALE of Aboriginal and Torres Strait Islanders. S4 Table was used to triangulate HALE, LE, and Quality of Life outcomes, enabling comparison of regional-level estimates where available. Findings from scientific papers and government reports were compared side-by-side to highlight areas of convergence, divergence, and evidence gaps. This synthesis informed the structuring of the results section and the identification of priority areas for further research and policy attention. The results were summarised narratively and organised thematically according to the key domains identified in the review. Data extraction was managed in Microsoft Excel, while thematic analysis of qualitative findings was undertaken using NVivo 14 (QSR International). This software facilitated systematic coding and organisation of extracted data, supporting the identification of key themes and patterns.

**Table 2:** Summary characteristics of the peer-reviewed studies included in the scoping review.

SL#	Author(s), Year	Study Design	Method of the study	Study Population /Participants	Age of the study participants	Study period	Sample size	Study setting
1	Gall et al., 2021	Cross-sectional study	Quantitative	Aboriginal and Torres Strait Islander	18 and above	2019-2020	663	National
2	Dawson et al., 2023	Cohort study	Quantitative	Aboriginal and Torres Strait Islander and non-Indigenous populations	18 and above	2019-2020	3890	Victoria
3	Li & McDermott, 2017	Cohort study	Quantitative	Aboriginal and Torres Strait Islander	15 and above	1998-2000	2787	North Queensland
4	Coombes et al., 2018	Descriptive study	Qualitative	Aboriginal and Torres Strait Islander	45–89	Not specified	60	New South Wales
5	Davey, 2021	Descriptive Analytical Study	Quantitative (Secondary data review)	Aboriginal and Torres Strait Islander and non‑Indigenous Australians	Not specified	2018–2020	Not specified	Northern Territory
6	Zhao et al., 2022	Retrospective Trend Analysis	Quantitative	Aboriginal and Torres Strait Islander and non‑Indigenous Australians	All age groups	1999–2018	Not specified	Northern Territory
7	Gardiner et al., 2021	Incidence population-based study (Secondary study data sourced from ABS and National Health Survey)	Quantitative	Aboriginal and Torres Strait Islander and non‑Indigenous Australians	Not specified	2019-2020	Not specified	National
8	Randall et al. 2018	Cohort study	Quantitative	Aboriginal and Torres Strait Islander and non‑Indigenous Australians	All age groups	2003 and 2013	5437018	New South Wales
9	Zhao et al., 2024	Cross-sectional study	Quantitative	Aboriginal and Torres Strait Islander and non‑Indigenous Australians	All age groups	2014 and 2018	5593 deaths and 520,195 hospitalisation	Northern Territory
10	Wettasinghe et al., 2020	Participatory action research	Qualitative	Aboriginal and Torres Strait Islander	50 years and above	2014 and 2017	34 Participants	New South Wales
11	Banham et al., 2019	Cross-sectional study	Quantitative	Aboriginal and Torres Strait Islander	15 years and above	2010−11	399	South Australia

**Table 3:** Background of the government reports included in the review.

SL#	Report Issuing Authority	Name of the Report/Web Article	Year of Publication	Population Covered	Outcome reported
1	ABS, 2023	Understanding life expectancy estimates for 2020–2022	2023	Aboriginal and Torres Strait Islander population	Life Expectancy at Birth (by sex and state/territory)
2	AIHW, 2022	Australian Burden of Disease Study 2018: Impact and Causes of Illness and Death in Aboriginal and Torres Strait Islander People	2022	Aboriginal and Torres Strait Islander population	Burden of Disease (DALY): Indigenous Australians experience a much higher overall burden of disease than non-Indigenous Australians, mainly from chronic conditions like heart disease, diabetes, kidney disease, and respiratory illness. Health-Adjusted Life Expectancy (HALE): Indigenous Australians live fewer years in good health and spend more years with illness or disability compared to non-Indigenous people. Cause-specific Burden: The largest contributors to poor health were mental and substance use disorders, injuries, cardiovascular disease, diabetes, and cancer. Comparative Outcomes: Both earlier deaths and longer periods of ill health explain the health gap between Indigenous and non-Indigenous Australians.
3	AIHW, 2024	Determinants of health for Aboriginal and Torres Strait Islander people	2024	Aboriginal and Torres Strait Islander population	Social determinants such as education, income, employment, housing, food security, and experiences of racism were strongly linked to health outcomes, including life expectancy, mental health, and chronic conditions. The findings showed a clear social gradient in health across Socio-Economic Indexes for Areas (SEIFA) quintiles, with health outcomes improving as socio-economic advantage increased. In addition, regional inequalities persisted, with Aboriginal and Torres Strait Islander people in remote areas experiencing poorer health outcomes than those in metropolitan regions.
4	Australian Government & Productivity Commission, 2025	Closing the Gap Annual Data Compilation Report 2025	2025	Aboriginal and Torres Strait Islander population	Progress towards the 17 socio-economic outcome targets under the National Agreement on Closing the Gap. These targets spanned areas such as life expectancy, child development, employment, housing, justice, health, and digital inclusion. The report showed that while some improvements had been made, significant gaps remained in achieving equity between Aboriginal and Torres Strait Islander people and the broader Australian population.
5	AIHW, 2017	Trends in Indigenous mortality and life expectancy 2001–2015	2017	Aboriginal and Torres Strait Islander people	Between 2001 and 2015, life expectancy at birth for Indigenous Australians increased by around 4 years for males and 2.5 years for females. Over the same period, the life expectancy gap between Indigenous and non-Indigenous Australians narrowed slightly for males but showed little change for females.
6	AIHW, 2025	Queensland Report on Indigenous Health Performance Framework 2025	2025	Aboriginal and Torres Strait Islander people in Queensland	The Performance Framework (2025) assessed 68 indicators of health status, social and cultural determinants, and health system performance for Aboriginal and Torres Strait Islander people. The report showed persistently higher rates of chronic diseases such as diabetes, kidney disease, and cardiovascular disease, along with greater exposure to social disadvantage including lower income, poorer housing conditions, and higher unemployment compared to the non-Indigenous population. It also identified regional disparities, with Indigenous Queenslanders in remote and very remote areas experiencing significantly poorer health outcomes and more limited access to preventive and specialist services than those in metropolitan regions.
7	AIHW, 2025	Health and wellbeing of First Nations people	2025	Aboriginal and Torres Strait Islander people	Nearly half of First Nations people rated their health as fair or poor, with long-term conditions such as diabetes, cardiovascular disease, and kidney disease contributing substantially to this outcome. Mental health issues were also significant, with high levels of psychological distress and a suicide rate almost twice that of the non-Indigenous population. Mortality trends showed some improvement over time, but the leading causes of death continued to include cardiovascular disease, cancer, and injuries. Burden of disease estimates highlighted that First Nations people experienced a disproportionate share of disability-adjusted life years (DALYs), particularly years of life lost (YLL) due to premature mortality and years lived with disability (YLD) linked to chronic conditions and mental health problems.
8	AIHW, 2021	Older Australians – AIHW 2021	2021	Aboriginal and Torres Strait Islander population and non-Indigenous population in Australia	Chronic conditions were common, with cardiovascular disease, diabetes, chronic respiratory disease, and kidney disease contributing to multimorbidity. Disability prevalence was higher among older Aboriginal and Torres Strait Islander people compared to their non-Indigenous peers For older Aboriginal and Torres Strait Islander Australians, the main causes of death were conditions affecting the heart and blood flow, problems related to breathing, diabetes, and some types of cancer. Although life expectancy at older ages had improved over time, the gap with non-Indigenous Australians remained, reflecting earlier and heavier health problems across the life course.
9	AIHW, 2024	Socioeconomic Indexes (Measure 2.09) – AIHW Indigenous HPF	2024	Aboriginal and Torres Strait Islander people	Distribution of the Indigenous population across SEIFA quintiles; proportion of people in most disadvantaged quintiles by state; implications for access to services, health, education, and employment outcomes
10	AIHW, 2024	Size and sources of the health gap for Australia’s First Nations people: 2017–2019	2024	Aboriginal and Torres Strait Islander and non-Indigenous Australians	Aboriginal and Torres Strait Islander people live about 10 fewer healthy years than non-Indigenous Australians result of the overall burden of disease. About 35% of this health gap is explained by poorer access to education, jobs, income, and housing among Indigenous people compared with non-Indigenous people. Another 30% is due to higher smoking, obesity, and alcohol use in Indigenous communities. The biggest gaps are seen in heart disease, diabetes, and suicide, and these are often worse in remote areas than in cities among the Aboriginal population than the non-Indigenous. Indigenous Australians make fewer visits to psychologists and GPs for mental health support compared with non-Indigenous Australians, with the lowest access in remote communities.
11	AIHW, 2023	Access to mental health services (Measure 3.10, Indigenous HPF)	2023	Aboriginal and Torres Strait Islander people	Aboriginal and Torres Strait Islander people made increasing use of Indigenous-specific Medicare mental health services, including visits to psychologists and general practitioners. However, substantial geographic variation was evident, with people in remote areas having less access to these services than those in urban locations. Despite improvements, Aboriginal and Torres Strait Islander people continued to access mental health services at lower rates than non-Indigenous Australians.

## Results

### Search results

The search identified 2,395 records from database searches and 13 additional records through hand-searching and grey literature sources. After removing 161 duplicates, 2,247 titles and abstracts were screened. Of these, 220 full texts were assessed for eligibility, with 193 excluded based on the pre-specified criteria. Five could not be retrieved in full. A total of 22 sources met the inclusion criteria, comprising 11 peer-reviewed scientific papers (Table 2) and 11 government reports (Table 3). The study selection process is illustrated in the PRISMA-ScR flowchart [18] (Fig 1).

**Fig 1:**
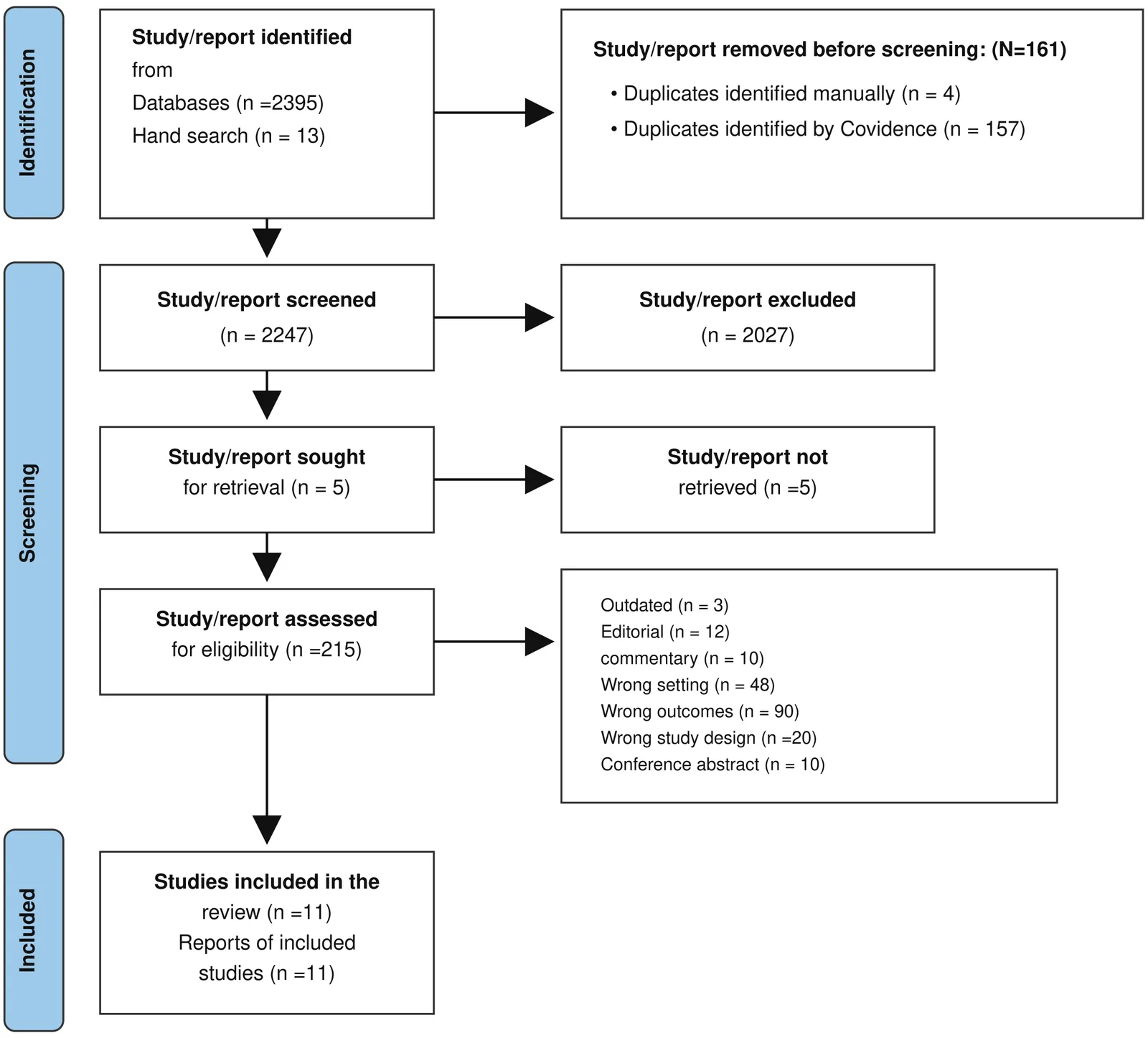
PRISMA Flow Chart for the Selection and Screening Process of Studies Included in the Scoping Review.

### Background of Included Sources

#### Peer-reviewed papers.

The 11 peer-reviewed studies (Table 2), published between 2017 and 2024, included nine quantitative [2,19,20] and two qualitative [21,22] studies. Five studies [20–24] involved Aboriginal and Torres Strait Islander populations exclusively, while six [2,19,25–27] included both Indigenous and non-Indigenous groups. Regarding age, two studies [20,27] included adults aged 18 years and above, three studies [2,19,28] covered all ages, and two [21,22] focused on older Aboriginal people aged 50 years and above. Geographically, two studies [20,25] were conducted at the national level, three studies [2,26,28] in the Northern Territory (Peer-reviewed), three in New South Wales (NSW) [19,21,22], one in Victoria [27], one in South Australia (SA) [23], and one [24] in North Queensland (QLD). Sample sizes ranged from 34 participants in a qualitative study to 5,437,018 records in a population-based cohort.

### Government reports

The 11 government reports (Table 3), published between 2017 and 2025, included nine by AIHW [4,7,14,29], one by ABS [30], and one jointly by the Australian Government and the Productivity Commission [31]. Data sources included the ABS Census of Population and Housing (n = 5) [1,7,29,30,32], National Aboriginal and Torres Strait Islander Health Survey (NATSIHS, n = 5) [14,30–33], National Aboriginal and Torres Strait Islander Social Survey (NATSISS, n = 3) [1,14,33], National Mortality Database (n = 7) [1,7,29–34], and linked hospitalisation and primary care records (n = 5) [1,7,30–34]. Outcomes reported included Life Expectancy (LE, n = 3) [29,30,32], Healthy Life Expectancy (HALE, n = 1) [7], burden of disease estimates (n = 4) [1,7,31,32], chronic condition prevalence (n = 6), mental health outcomes (n = 4) [4,32,34,35], and socio-economic indicators (n = 7) [1,4,14,30–35].

### Thematic review

Fig 2 visualises the key themes emerging from this scoping review. Four interlinked domains were identified as influencing HALE among Aboriginal and Torres Strait Islander peoples: physical chronic conditions, mental health, socio-economic factors, and regional disparities affecting Aboriginal and Torres Strait Islanders. These domains are closely interconnected, with physical and mental well-being shaped by socio-economic realities and further influenced by geographic and demographic context. Collectively, these findings highlight how multiple, overlapping determinants contribute to disparities in both the length and quality of life in Indigenous populations.

**Fig 2:**
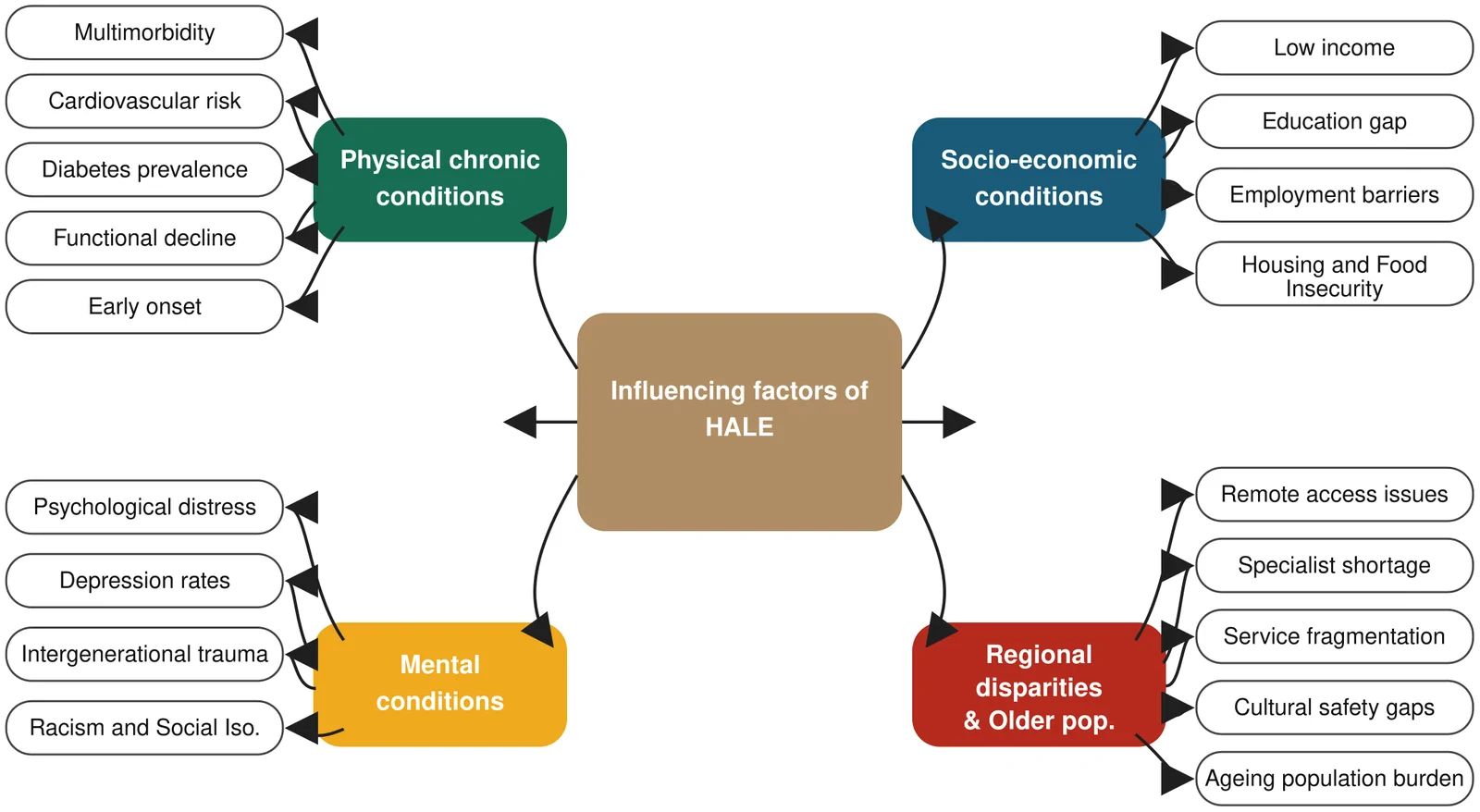
Key Factors Influencing HALE among Aboriginal and Torres Strait Islander People.

Within the reviewed literature, only two government reports explicitly reported HALE alongside LE for Aboriginal and Torres Strait Islander populations [7,30]. Among peer-reviewed sources, Zhao et al. (2024) presented HALE and Unhealthy Life Expectancy estimates for Indigenous and non-Indigenous males and females in the Northern Territory. Zhao et al. (2022) further reported LE for both groups in the Northern Territory [2,28]. Although the included studies did not provide comprehensive estimates of HALE or LE across Australian states and territories, they did offer clear descriptions of socioeconomic, physical, mental health, and regional determinants that could contribute to differences in HALE/LE between Indigenous and non-Indigenous populations.

### Physical health conditions

Chronic conditions consistently emerged as the principal drivers of reduced LE and HALE among Aboriginal and Torres Strait Islander peoples. National data (S3 Table) highlight coronary heart disease, diabetes, chronic obstructive pulmonary disease (COPD), kidney disease, and cancers as the most significant contributors to the burden of disease [4,7,34,35]. Among those aged 65 years and above, these conditions, along with dementia, accounted for an estimated 240,000 years of healthy life lost, equivalent to 289 years per 1,000 people [7]. Mortality statistics reinforce this disparity, with diabetes-related deaths more than five times higher and injury and poisoning nearly twice as high among Indigenous Australians compared with their non-Indigenous counterparts [32].

Peer-reviewed studies (S2 Table) reveal that such conditions not only occur more often but also emerge earlier in life. Randall et al. (2018) demonstrated that multimorbidity was more than twice as common among Aboriginal people, appearing from as early as age 25 and widening across midlife [19]. This early onset sets the stage for diminished health in later years. Gall et al. (2021) extended this evidence by linking multimorbidity to subjective well-being, showing that individuals with seven or more conditions rated their well-being scores eight points lower than those without illness [20]. Banham et al. (2019) reinforced this pattern, documenting a stepwise decline in self-rated health as the number of chronic conditions increased, with the steepest drop among those reporting three or more [23]. Chronic illness therefore appears to reduce well-being well before older age.

The survival implications of these health burdens are further evident in biological and clinical studies. Li and McDermott (2017) found that markers of chronic disease, including high blood sugar and early kidney damage, were strongly associated with prolonged periods of poor health and diminished quality of life [24]. Cardiovascular studies add further concern. Gardiner et al. (2021) reported that serious cardiac events occur at younger ages among Indigenous patients, exacerbated by limited access to diagnostic and specialist care [25]. Similarly, Dawson et al. (2023) demonstrated that Indigenous patients hospitalised for a heart attack in Victoria were not only to present with comorbid diabetes and kidney disease but also less likely to receive guideline-based treatment [27]. This pattern suggests that inequitable healthcare access compounds the effects of chronic disease on HALE.

Recent work has begun to quantify years lived in poor health. Zhao et al. (2024) identified diabetes, musculoskeletal disorders, and cardiovascular disease as leading contributors to reduced healthy life years, although the analysis did not disaggregate by age or region [2]. Complementary qualitative research provides further depth in understanding the lived experiences behind these estimates. Coombes et al. (2018) and Wettasinghe et al. (2020) described how chronic illness limited independence, reduced mobility, and increased care giving burdens within families [21,22]. These lived experiences mirror the diminished well-being and heightened mortality reported in quantitative studies but remain localised and not generalisable. Zhao et al. (2022) further identified dementia as a growing late-life contributor, particularly among Indigenous women [28]. These findings point to older age as an emerging focus for HALE research, though the precise number of healthy years lost to these conditions remains unclear.

### Mental health conditions

Mental health conditions consistently emerge as major contributors to the disease burden among Aboriginal and Torres Strait Islander peoples (S3 Table). Estimates from AIHW (2022) showed that anxiety (23%), alcohol use (19%), and depressive disorders (19%) were the leading causes of mental and substance use disorders, together accounting for nearly one-quarter of the total disease burden and 42% of the non-fatal burden in 2018 [7]. The Productivity Commission (2025) confirmed the same proportional contributions, indicating that despite ongoing policy efforts, the leading mental health concerns have remained unchanged [31].

Surveillance data from AIHW (2025) further quantified the scale of mental health challenges. It found that 31% of First Nations adults aged 18 years and over reported high or very high levels of psychological distress. The same report recorded a suicide rate of 26 per 100,000 population between 2015 and 2019, with higher rates among males (39 per 100,000) than females (13 per 100,000) [32]. Additional monitoring in 2024 revealed that almost one in four adults had been diagnosed with a mental health condition, while suicide had become a leading cause of death among children and young people, occurring at more than twice the rate of their non-Indigenous peers [34]. While these reports establish the overall scale of the mental health burden, they provide limited age-specific insights, particularly for older populations, leaving a gap in understanding how mental health conditions shape Healthy Life Expectancy across different stages of life [4].

Peer-reviewed studies (S2 Table) provide additional context on the mental health burden and its implications for HALE. Gall et al. (2021) reported that individuals with a diagnosed mental health condition had well-being scores approximately 12 points lower than those without such conditions [20]. Coombes et al. (2018) contributed a qualitative perspective, describing how depression and isolation among Aboriginal elders fostered feelings of despair, hopelessness, and a perception of “waiting for death,” which hindered engagement in health improvement initiatives [22]. This qualitative account offers some insight into the older-age gap noted above, though it is based on a small, localised sample, which limits generalisability to broader populations.

Emerging evidence points to age-specific trends, though results are uneven. Zhao et al. (2022) reported dementia as a leading cause of poor health before death for Indigenous women and men [28]. Zhao et al. (2024) further estimated that mental and substance use disorders accounted for an additional 3.4 years lived in poor health for males and 3.1 years for females, directly contributing to the gap in healthy life expectancy [2]. Yet, both studies treated mental illness primarily as an aggregate burden, leaving cultural and psychosocial contexts unexplored. Randall et al. (2018) offered a contrasting perspective, finding that younger adults reported a higher prevalence of mental health problems compared with older groups [19]. This suggests either greater resilience or under-reporting among elders, but the mechanisms remain unclear. The psychosocial dimensions of mental health also received attention. Wettasinghe et al. (2020) linked depression to grief, isolation, and the intergenerational trauma of the Stolen Generations, showing how structural and historical factors intensify risks among Aboriginal and Torres Strait Islander communities [21]. Stigma and shame were identified as further barriers to seeking care, highlighting systemic challenges that are not captured in national reporting. Davey. (2020) explored the relationship between mental illness and suicide among young people, while noting that age-disaggregated evidence remains limited for other groups [26]. These studies highlight the importance of contextual and cultural dimensions that government reports often overlook [25].

### Socio-economic factors

Government reports (S3 Table) consistently identified socio-economic disadvantage as a key driver of poorer health outcomes and reduced LE among Aboriginal and Torres Strait Islander peoples. Estimates from AIHW (2022) indicated that housing, education, and income contribute indirectly to disease burden through risk factor attribution [7]. National analyses by AIHW (2024) and the Productivity Commission (2025) estimated that social determinants explained 35% of the health gap, followed by employment (14.4%), income (12.6%), and school completion (8.9%) [31,35]. This report also showed that employed adults had 1.5 times higher odds of good health than the unemployed, and Year 12 completion increased the odds by 1.3 times. Together, these reports quantify the contribution of social determinants but do not examine how these risks shape HALE across the life course, particularly for older age groups [1].

Across peer-reviewed studies (S2 Table), socio-economic position was consistently linked with poor health outcomes and lower well-being among Aboriginal and Torres Strait Islander peoples. Gall et al. (2021) found a strong association between socio-economic and household factors and well-being. For example, individuals who had completed Grade 12 reported scores about 8 points higher on the Visual Analogue Scale (0–100) than those with Grade 10 or below. The study also reported that those in full-time employment had well-being scores 7 points higher than those not working, and 6 points higher than those in part-time employment. Living without a partner was associated with a 5-point lower well-being score compared with those living with a partner. Larger households (four or more members) were associated with 8–9 points higher well-being compared to single-person households. Furthermore, the study also found that financial strain reduced well-being, with those reporting ‘not enough money’ scoring eight points lower and those with ‘just enough’ scoring five points lower than Indigenous people with financial security [20]. However, the study did not assess whether these effects on well-being translate into differences in HALE across age groups.

Age-related disparities offered further insight into how socio-economic disadvantage shapes health trajectories over time. Dawson et al. (2023) showed that Aboriginal patients presented with heart attacks at younger ages than non-Indigenous patients, suggesting that disadvantage accelerates disease onset [27]. Coombes et al. (2018) described barriers faced by older Aboriginal people including lack of transport, financial strain, and limited culturally safe services, which restricted their ability to engage with care [22]. These individual barriers were reinforced by community-level findings from Davey (2020), who demonstrated that overcrowded housing, poverty, and remoteness further increased infection risks and limited healthcare access [26]. Gardiner et al. (2021) found that socio-economic disadvantage also contributed to delays in diagnosis and treatment in both remote and urban contexts [25]. Collectively, these studies illustrate how disadvantage operates at individual, community, and clinical settings, though none explicitly examined how these experiences reduce HALE.

Quantitative evidence (S2 Table) further confirmed the depth of these disparities. Randall et al. (2018) reported that 55% of Aboriginal patients lived in the lowest socio-economic quintile and that disadvantage was more strongly associated with multimorbidity and mortality than rurality [19]. Wettasinghe et al. (2020) showed how disadvantage translated into practical barriers such as low health literacy, stigma, and lack of transport, which restricted program access [21]. Banham et al. (2019) similarly found lower health utility among women, older adults, and the underemployed [23]. This body of evidence shows that socio-economic disadvantage undermines well-being through both clinical outcomes and access barriers, though none explicitly examined how these disadvantages reduce HALE.

### Regional factors

Government reports (S3 Table) frequently highlighted substantial geographic disparities in health outcomes among Aboriginal and Torres Strait Islander peoples. Analyses from AIHW (2025) and the Productivity Commission (2025) consistently showed that residents in remote and very remote areas faced higher burdens of chronic disease, injury, and mortality than those in regional or metropolitan locations [31,32]. For example, First Nations males in remote areas had a life expectancy of 67.3 years compared to 72.5–72.8 years in major cities and regional areas [31]. Similarly, mortality rates for conditions such as diabetes were up to 5.6 times higher among Indigenous Queenslanders compared with other non-Indigenous counterparts, with the disparity particularly pronounced in remote communities [31].

This review further identified that eight of the eleven included papers (S2 Table) reported stand-alone state or regional analyses [2,19–28]. The two national studies offered no sub-state breakdown [20,25]. Each paper described conditions within its own setting. Most studies measured prevalence or service access rather than long-term outcomes [2,19,20], and few provided age-specific analyses, limiting insight into how regional disadvantage influences HALE across different life stages [21,22].

## Discussion

This review found that HALE among Aboriginal and Torres Strait Islander peoples is influenced by the combined effects of socioeconomic, physical, mental health, and regional determinants. Disadvantage arises from the cumulative impact of structural inequities, chronic disease burdens, and geographic isolation that reinforce one another over the life course. Evidence generated from quantitative studies, qualitative research, and national government reports, the review explored consistent patterns across multiple data sources. These findings directly address the review questions by demonstrating how physical, mental, socio-economic, and regional factors interact to shape HALE outcomes. The results are particularly relevant for Aboriginal and Torres Strait Islander communities, as well as policymakers and health system stakeholders aiming to reduce inequities in healthy life expectancy.

Socioeconomic disadvantage remains one of the most consistent and powerful determinants of reduced HALE among Aboriginal and Torres Strait Islander peoples. We found that lower income, educational attainment, and employment rates are systematically linked with earlier onset of chronic conditions and higher disability burden [20,31,36]. These socioeconomic inequities interact with structural barriers to healthcare access, such as service availability, affordability, and cultural safety, to amplify disparities in HALE [37,38]. International research on Indigenous populations in Canada and New Zealand mirrors these findings, demonstrating that material deprivation and educational disadvantage translate into shorter lives lived in good health, even when healthcare is universal [39,40]. The convergence of evidence across contexts reinforces the interpretation that reducing socioeconomic inequality is central to narrowing HALE gaps, not only by targeting income and employment, but also by addressing the intergenerational impacts of poverty on health behaviours, stress, and access to preventive care.

Physical health disparities, particularly the high prevalence of preventable chronic diseases, are a key driver of the HALE gap between Indigenous and non-Indigenous Australians [7]. Peer-reviewed studies corroborate these trends, highlighting earlier onset and higher multimorbidity rates that reduce the proportion of life spent in good health [2,23]. International literature adds weight to this interpretation, with similar patterns documented among Māori in New Zealand and First Nations populations in Canada, where preventable diet- and lifestyle-related diseases are major drivers of reduced quality-adjusted life years [41,42]. This alignment across national and global evidence strengthens the argument that addressing upstream determinants such as diet, physical activity, and access to timely diagnosis is essential for extending both lifespan and health span. Furthermore, evidence generated from the reviewed literature also echoes with broader global Indigenous health literature, where premature onset of multimorbidity is common, reducing the proportion of life lived in good health and thereby lowering HALE [42,43]. This pattern emphasises the need for preventive approaches targeting early and mid-life stages, particularly given that much of the morbidity burden is potentially avoidable through improved primary care, culturally safe screening, and integrated chronic disease management with direct implications for extending HALE. Yet, physical health alone does not fully explain reduced HALE; mental health plays an equally critical role.

Mental health also plays a critical role in shaping HALE outcomes, interacting closely with physical and social disadvantage. National data show a higher prevalence of psychological distress, depression, and anxiety among Aboriginal and Torres Strait Islander peoples compared with non-Indigenous Australians [34], with comorbidity between mental and physical illness exacerbating functional decline and reducing years lived in good health [20]. These burdens are deeply rooted in historical and contemporary determinants, including intergenerational trauma, racism, and social exclusion [44,45], which shape help-seeking behaviour and limit access to culturally safe care evidence from other Indigenous contexts strengthens this interpretation. Studies in the United States and Canada show that mental health acts as a mediator between social disadvantage and reduced health-adjusted life expectancy [46,47]. The Maori experience in Aotearoa/New Zealand adds another dimension, demonstrating that when psychological well-being is addressed alongside physical health, Indigenous populations experience measurable gains in health-adjusted survival, with particular benefits observable among older cohorts [48]. The evidence collectively indicates that mental health is not secondary to physical illness, but a core dimension of HALE requiring integrated and culturally responsive approaches to health promotion and service delivery. Geographic location adds another critical layer of influence on HALE outcomes for Aboriginal and Torres Strait Islander peoples, intersecting with socio-economic, physical, and mental health disadvantages. Although some studies and reports present state-level or remote/urban breakdowns, none provided HALE estimates across all Australian states and territories within a single analysis. This absence of nationally consistent, disaggregated data creates a critical gap for policy comparability and makes it difficult to monitor geographic equity in ageing outcomes [31].

Despite this limitation, the available evidence indicates clear disparities. Remote and very remote regions experience higher burdens of chronic disease and premature mortality, largely driven by reduced service accessibility, workforce shortages, and entrenched socio-economic disadvantage [7,36]. These challenges are not unique to Australia; peer-reviewed literature documented similar rural health inequities among Indigenous populations in Canada, the United States, and New Zealand [39,49]. This international consistency reinforces the view that geography is a structural determinant of health, shaping opportunities for HALE across contexts.

Taken together, these findings show that physical, mental, socio-economic, and regional disadvantage do not operate independently but compound one another. Aboriginal and Torres Strait Islander people living in remote areas experience a higher burden of chronic disease [7,36]. They also face reduced access to culturally safe mental health care [22,26] concentrated socio-economic disadvantage [8,31,32] and structural barriers created by distance and workforce shortages [6]. While several individual studies and government reports touched on more than one of these domains, for example, five of the eleven included peer-reviewed studies [19–22,26] reported on physical, mental, and socio-economic outcomes together (S2 Table), none analysed how these domains interact and compound one another for the same population, nor combined this with a systematic regional comparison. This integrated, cross-domain analysis, generated only through synthesis across sources, is a distinct contribution of this review.

### Implications for research and policy

This review identifies critical evidence gaps in HALE measurement for Aboriginal and Torres Strait Islander peoples, with direct relevance for research and policy. The absence of nationally consistent, state-level data makes it difficult to design targeted interventions that respond to regional needs. Equally important is the lack of age-specific analyses, which restricts understanding of health trajectories across different life stages, including older populations. In addition, few studies have integrated physical, mental, and socio-economic determinants into HALE measurement, limiting insight into how these domains interact to shape well-being across the life course. Future research should therefore prioritise the standardisation of HALE measures, including the use of culturally sensitive age thresholds, and adopt more holistic approaches that reflect the multidimensional nature of health. Embedding Indigenous leadership in program design and ensuring regional tailoring of services are also crucial steps toward closing the HALE gap. Together, these priorities call for a shift away from a one-size-fits-all approach toward policies that reflect cultural contexts, regional differences, and the lived experiences of Aboriginal and Torres Strait Islander peoples.

### Strengths and limitations

A key strength of this review is its comprehensive and innovative approach. To the best of our knowledge, this is the first synthesis to systematically integrate government reports alongside peer-reviewed studies in examining HALE and related outcomes among Aboriginal and Torres Strait Islander populations. Including these grey literature sources enabled us to capture recent, policy-relevant, population-level data that are often missing from academic publications. This approach aligns with recommendations for enhancing the comprehensiveness of Indigenous health reviews [50]. The review also applied a rigorous, transparent methodology aligned with the PRISMA-ScR framework [17], ensuring reproducibility and methodological integrity. Furthermore, thematic synthesis enabled triangulation across multiple data types and contexts, strengthening the interpretive validity of our findings.

This review also demonstrates a strength in scope and inclusivity. By not restricting the analysis solely to disease-specific outcomes, it considered a broader range of health determinants (socio-economic, physical, mental, and geographic) in line with the holistic conceptualisations of health used in Indigenous health frameworks (National Aboriginal Community Controlled Health Organisation) [51].

However, certain limitations should be acknowledged. First, critical appraisal of individual sources was not conducted. As a result, the methodological rigour of individual sources, including some government reports that lacked detailed methodological information, could not be systematically evaluated. This should be considered when interpreting the findings. Second, the review identified a lack of consistent state-level disaggregation for HALE or Quality of Life; most reports presented results for a single state or grouped two to three states, rather than providing comprehensive national breakdowns. Furthermore, restricting the review to English-language publications and studies published from 2015 onwards may have excluded relevant evidence and limited the capture of historical perspectives and long-term trends in Aboriginal and Torres Strait Islander HALE and well-being.

## Conclusion

Socio-economic disadvantage, preventable chronic disease, and high levels of psychological distress were consistently associated with reduced HALE, while geographic isolation further intensified these risks through barriers to timely and culturally safe care. Although some studies provided state or region-specific data, there was limited nationally consistent evidence on HALE, and few analyses disaggregated results for the oldest age cohorts. Furthermore, interactions between physical, mental, and socio-economic factors were rarely investigated together, leaving the cumulative effects on HALE outcomes underexplored. Addressing these evidence gaps will require studies that integrate multiple determinants, incorporate age-specific analysis, and generate regionally inclusive estimates. Such research would strengthen the evidence base needed to inform policies and programs aimed at reducing inequities in HALE across the life course, with particular attention to later-life challenges among Aboriginal and Torres Strait Islander peoples.

## Supporting information

S1 Table Search terms applied across each database. (DOCX)

S2 Table Summary of findings from included peer-reviewed studies on Physical, Mental, and Socio-economic Determinants of Health Outcomes among Aboriginal and Torres Strait Islander Peoples. (DOCX)

S3 Table Summary of Findings from Government Reports on Physical, Mental, and Socio-economic Determinants of Health Outcomes among Aboriginal and Torres Strait Islander Peoples. (DOCX)

S4 Table Summary of Government Reports and Scientific Papers on HALE, LE, and Quality of Life. (DOCX)

S1 Checklist Preferred Reporting Items for Systematic reviews and Meta-Analyses extension for Scoping Reviews (PRISMA-ScR) Checklist. (DOCX)
