# Supplementary material for: Understanding the factors shaping Healthy Life Expectancy (HALE) in Indigenous Australians: A Scoping Review
Source: PLoS One. 2026 Nov 11;21(11):e0347718. doi: 10.1371/journal.pone.0347718 (PMC13754099; doi:10.1371/journal.pone.0347718)
Supplement: Supplementary file 1 [file pone.0347718.s001.docx]

**S1 Table. Search terms applied across each database**

| Name of database | Search term used | limit |
| --- | --- | --- |
| Scopus | TITLE-ABS-KEY("Healthy life expectancy" OR "Life Expectancy" OR "Quality of Life" OR "Functional life expectancy") AND ("indigenousness" OR "aboriginal" OR "first nation" OR "Torres Islander") AND ("Australia") | English language  Year range: 2015-2025 |
| Web of Science | Topic (TS) ("Healthy life expectancy" OR "Life Expectancy" OR "Quality of Life" OR "Functional life expectancy") AND ("indigenousness" OR "aboriginal" OR "first nation" OR "Torres Islander") AND ("Australia") | English language  Year range: 2015-2025 |
| Chinhal | ("Healthy life expectancy" OR "Life Expectancy" OR "Quality of Life" OR "Functional life expectancy") AND ("indigenousness" OR "aboriginal" OR "first nation" OR "Torres Islander") AND Australia | English language  Year range: 2015-2025 |
| PsycINFO | ("Healthy life expectancy" OR "Life Expectancy" OR "Quality of Life" OR "Functional life expectancy").mp. AND ("indigenousness" OR "aboriginal" OR "first nation" OR "Torres Islander").mp. AND Australia.mp  [mp=title, abstract, heading word, table of contents, key concepts, original title, tests & measures, mesh word] | English language  Year range: 2015-2025 |
| Science direct | ("Healthy life expectancy" OR "Life Expectancy" OR "Quality of Life" OR "Functional life expectancy")AND ("indigenousness" OR "aboriginal" OR "first nation" OR "Torres Islander") AND Australia | English language  Year range: 2015-2025 |
| Medline | ("Healthy life expectancy" OR "Life Expectancy" OR "Quality of Life" OR "Functional life expectancy").mp. AND ("indigenousness" OR "aboriginal" OR "first nation" OR "Torres Islander").mp. AND Australia.mp  [mp=title, abstract, heading word, table of contents, key concepts, original title, tests & measures, mesh word] | English language  Year range: 2015-2025 |
| Social science database | ("Healthy life expectancy" OR "Life Expectancy" OR "Quality of Life" OR "Functional life expectancy") AND ("indigenousness" OR "aboriginal" OR "first nation" OR "Torres Islander") AND Australia | English language  Year range: 2015-2025 |
| Google scholar | ("Healthy life expectancy" OR "Life Expectancy" OR "Quality of Life" OR "Functional life expectancy") "indigenousness" OR "aboriginal" OR "first nation" OR "Torres Islander" AND Australia | English language  Year range: 2015-2025 |
