# Supplementary material for: Understanding the factors shaping Healthy Life Expectancy (HALE) in Indigenous Australians: A Scoping Review
Source: PLoS One. 2026 Nov 11;21(11):e0347718. doi: 10.1371/journal.pone.0347718 (PMC13754099; doi:10.1371/journal.pone.0347718)
Supplement: Supplementary file 2 [file pone.0347718.s002.docx]

**S2 Table. Summary of findings from included peer-reviewed studies on Physical, Mental, and Socio-economic Determinants of Health Outcomes among Aboriginal and Torres Strait Islander Peoples**

| **SL#** | **Name of study** | **Physical Chronic Conditions and Health Outcomes** | **Mental Health Conditions and Health Outcomes** | Socio-economic Conditions and Health Outcomes | Study settings |
| --- | --- | --- | --- | --- | --- |
| 1 | Gall et al., 2021 | Aboriginal and Torres Strait Islander population with seven or more (≥7) physical comorbidities had wellbeing scores approximately 8 points lower ^[1]^ on the well-being scale compared to those with no comorbidities | Aboriginal and Torres Strait Islander peoples with any mental health condition had well-being about 12 points lower than those with no mental health condition. | - Aboriginal and Torres Strait Islander adults aged 30–44 reported well-being scores about 5 points higher than those aged 45–59, while those aged 60 and above reported scores 11 points higher than the 45–59 age group. - Single Aboriginal and Torres Strait Islander participants reported well-being scores 5 points lower than partnered individuals. - Living in larger households was associated with better outcomes; households of four reported scores 9 points higher and households of five or more 8 points higher compared with sole occupiers. - Aboriginal and Torres Strait Islander people who had completed Grade 12 reported well-being scores 8 points higher than those with Grade 10 or below. - Aboriginal and Torres Strait Islander people who were not working reported well-being scores 7 points lower than those in full-time work, while part-time workers reported scores 6 points lower, and those in other employment arrangements reported scores 10 points lower. - Financial security was a key determinant: participants reporting “not enough money” scored 8 points lower, and those with “just enough” scored 5 points lower than those with more than enough. | National level study |
| 2 | Dawson et al., 2023 | Indigenous Australians experienced disproportionately higher prevalence of diabetes, chronic kidney disease, prior coronary disease, and chronic airways disease compared with their non-Indigenous counterparts | Not Specified | Indigenous people with lower SES were affected by the early onset of diseases (diabetes, chronic kidney disease, prior coronary disease, and chronic airways disease) as compared to non-Indigenous people (Mean age of disease commencement:49.5 years for Indigenous and 62.7 years for non-Indigenous).Indigenous patients were less likely to follow recommended care protocols both before hospital admission (e.g., timely help-seeking, medication use) and during hospital treatment (e.g., compliance with procedures, discharge planning). | Victoria |
| 3 | Li & McDermott, 2017 | - Indigenous people with diabetes (high blood sugar) were more likely to die earlier than non-Indigenous people. Having a fasting blood sugar level of 5.5 mmol/L or higher was linked to a 50% higher risk of death - Indigenous people with kidney damage also faced higher risks. Mild kidney damage (microalbuminuria) was linked to a 40% higher risk of death, while severe kidney damage (macroalbuminuria) was linked to a 160% higher risk of death as compared to non-indigenous people. - Indigenous people with liver stress (measured by gamma-glutamyl transferase levels of 50 IU or higher) had a 40% higher risk of death compared to non-indigenous. | Not Specified | Not Specified | North Queensland |
| 4 | Coombes et al., 2018 | Chronic illnesses such as cardiovascular disease, diabetes, respiratory disease, and renal disease limit independence and daily activities in older First Nations people and reduce quality of life and healthy ageing | - Aboriginal and Torres Strait Islander elders expressed feelings of despair, hopelessness, and a sense of waiting for death. - Depression and social isolation among Aboriginal and Torres Strait Islander elders hindered their efforts to improve health and well-being. | Aboriginal and Torres Strait Islander elders experienced barriers to health and well-being due to a lack of culturally appropriate transport, financial constraints, limited cultural safety, and the absence of supportive community networks. | New South Wales |
| 5 | Davey, 2021 | - In the Northern Territory, Aboriginal people experienced much higher rates of serious health conditions compared with non-Indigenous residents. The differences included heart disease at 260 times higher, chronic lung disease at 40 times higher, kidney failure at 30 times higher, diabetes at 10 times higher, and early heart attacks at 9 times higher, while lung cancer occurred at about twice the rate. | Psychosocial distress was a critical concern, with suicidal behaviour linked to mental illness being particularly prevalent among young Indigenous people. | - High levels of household overcrowding among Aboriginal and Torres Strait Islander people were linked to increased transmission of infectious diseases. - Poor housing conditions among Aboriginal and Torres Strait Islander communities increased the overall risk of illness. - Poverty and low socio-economic status among Aboriginal and Torres Strait Islander people limited access to healthcare, adequate nutrition, and preventive services. - Living in remote areas reduced Aboriginal and Torres Strait Islander people’s access to specialist healthcare services. | Northern Territory |
| 6 | Zhao et al., 2022 | Not specified | Dementia, including Alzheimer's disease, was the fifth most frequent specific cause of death for Indigenous women and the 12^th^ most frequent for Indigenous men. | No | Northern Territory |
| 7 | Gardiner et al., 2021 | - Indigenous patients with heart, stroke, and vascular disease (HSVD) had an earlier onset of illness, reduced healthy life, and experienced a higher burden of heart failure and acute coronary syndromes compared to non-Indigenous Australians. - Heart failure hospitalisation rates among Aboriginal and Torres Strait Islander people were 11 times higher in men and 23 times higher in women aged 20–39 compared with non-Indigenous Australians. - Diabetes, obesity, hypertension, chronic kidney disease, and coronary artery disease were identified as key risk factors contributing to poor cardiovascular outcomes among Aboriginal and Torres Strait Islander people. | No | - Socioeconomic disadvantage, including low education, financial instability, and unemployment, was linked to lower well-being and a higher risk of chronic diseases among Aboriginal and Torres Strait Islander people. - Limited access to diagnostic services reduced the likelihood of timely treatment for Aboriginal and Torres Strait Islander people. - Aboriginal and Torres Strait Islander people living in non-remote areas had fewer hospital admissions despite experiencing a high burden of disease, highlighting gaps in urban healthcare. - Aboriginal and Torres Strait Islander people in remote areas faced limited access to preventive and specialist cardiac care compared with those in urban and regional settings. | National |
| 8 | Randall et al. 2018 | - Multimorbidity was ≥10% higher in Aboriginal patients from age 25, and ≥20% higher between ages 40–79 compared to non-Aboriginal patients; - Aboriginal patients had a higher prevalence of all physical conditions; Physical morbidities were more common than mental morbidities, especially in older Aboriginal Australians. - More conditions strongly predicted higher mortality and poorer quality of life. | - Higher prevalence of mental health issues among 25-54-years than old Aboriginal people (more than 50 years). | - 55% of Aboriginal patients lived in the lowest socio-economic quintile - After adjusting for age, sex, and socio-economic status, the likelihood of multimorbidity among Aboriginal people in NSW was 2.6 times higher as compared to the non-Aboriginal people. | New South Wales |
| 9 | Zhao et al., 2024 | - Diabetes accounts for 3.3 extra years in poor health conditions for Indigenous males and 4.8 extra years in poor health for females compared to non‑Indigenous counterparts. - Joint, bone, and muscle problems (Musculoskeletal disorders) — account for 2.9 extra years in poor health for Indigenous males and 4.4 extra years for females as compared to non-Indigenous comparators - Cardiovascular diseases, such as heart attacks and strokes, explained 18–21% of the gap in healthy life expectancy between Indigenous and non-Indigenous Australians | - Mental and substance use disorders contributed substantially to unhealthy life expectancy among Indigenous Australians in the Northern Territory. Indigenous males lived 3.4 years in poor health due to these conditions, compared with 1.5 years for non-Indigenous males. Similarly, Indigenous females spent 3.1 years in poor health, compared with 1.3 years for their non-Indigenous counterparts. | Not specified | Northern Territory |
| 10 | Wettasinghe et al., 2020 | - Chronic diseases such as diabetes, cancer, cardiovascular problems, emphysema, and liver disease were more common among older Aboriginal Australians than among non-Indigenous people. These conditions were exacerbated by unhealthy behaviours, including smoking, heavy alcohol use, poor diet, and obesity, which in turn contributed to shorter and less healthy lives for Indigenous Australians. - Memory loss and dementia are becoming more frequent, with some Aboriginal people showing signs of dementia in their 50s when non-Indigenous people are usually still healthy - Falls, pain, and mobility loss reduce independence and quality of life among the Indigenous population than the non-Indigenous population. | - Depression among Aboriginal and Torres Strait Islander elders was closely linked to isolation, grief, and loss, often compounded by illness-related withdrawal. - Poor mobility, transport difficulties, and grief associated with bereavement, the Stolen Generations, and land loss further undermined health and well-being. - Mental health issues were not limited to elders but also affected younger generations within Aboriginal and Torres Strait Islander communities. - Many health concerns remained hidden due to shame and stigma, reducing opportunities for timely care and support. | - Low levels of health literacy among Aboriginal and Torres Strait Islander people negatively affected lifestyle choices and health behaviours. - Limited access to healthcare, caused by staff shortages, distance, and transport barriers, restricted timely care for Aboriginal and Torres Strait Islander communities. - Technology-related barriers reduced Aboriginal and Torres Strait Islander people’s ability to access health services. - Economic inequality restricted opportunities for Aboriginal and Torres Strait Islander people to receive necessary treatment. - Participation in health programs was hindered by barriers such as transport difficulties, shame, stigma, and feelings of hopelessness. | New South Wales |
| 11 | Banham et al., 2019 | - Aboriginal people of South Australia with one or two chronic conditions reported lower health utility^[2]^ scores than those with no conditions, while those with three or more conditions experienced even larger decreases in health utility, reflecting a sharper decline in health-related quality of life. | - Not Specified | - Aboriginal and Torres Strait Islander females reported health utility scores that were 3% lower than those of males. - Aboriginal and Torres Strait Islander people aged 35 years and above were associated with progressively lower health utility compared with those aged 15–34 years. - Employment at home or outside the home was linked with a 5% higher health utility compared to underemployment among Aboriginal and Torres Strait Islander people. - Living in regional or remote areas was associated with a lower likelihood of completing health-related quality of life assessments, which may lead to an underestimation of poor health-related quality of life in these groups. | South Australia |

1. In this paper, wellbeing was assessed using a 100-point Visual Analogue Scale (VAS), where 0 = worst imaginable wellbeing and 100 = best imaginable wellbeing. Scores are presented as points (0–100), not percentages or probabilities.
2. Health utility referred to a numerical measure of health-related quality of life (HRQoL), derived from the SF-6D instrument. Utility scores ranged from 0 (equivalent to death) to 1 (full health), with lower values indicating poorer perceived physical, social, and mental health
