# Supplementary material for: Understanding the factors shaping Healthy Life Expectancy (HALE) in Indigenous Australians: A Scoping Review
Source: PLoS One. 2026 Nov 11;21(11):e0347718. doi: 10.1371/journal.pone.0347718 (PMC13754099; doi:10.1371/journal.pone.0347718)
Supplement: Supplementary file 3 [file pone.0347718.s003.docx]

**S3 Table. Summary of Findings from Government Reports on Physical, Mental, and Socio-economic Determinants of Health Outcomes among Aboriginal and Torres Strait Islander Peoples**

| SL# | Name of study | Physical Chronic Conditions and Health Outcomes | Mental Health Conditions and Health Outcomes | Socio-economic Conditions and Health Outcomes | Study settings |
| --- | --- | --- | --- | --- | --- |
| 1 | AIHW, 2022 | - Among Aboriginal people, aged 65–74 represented only 3.4% of the population in 2018 but accounted for 9.4% of the total burden of chronic disease. This indicated that older Aboriginal adults carried a disproportionately high share of chronic health problems relative to their population size. Among Aboriginal and Torres Strait Islander people, coronary heart disease (CHD), chronic obstructive pulmonary disease (COPD), lung cancer, kidney disease, and diabetes were the leading contributors to the total burden of chronic disease. | - In 2018, mental and substance use disorders were responsible for 23% of the total disease burden among Indigenous Australians. - Mental and substance use disorders also contributed 42% of the non-fatal burden experienced by Indigenous Australians in 2018. - The leading sub-causes of the burden from mental and substance use disorders were anxiety disorders (23%), alcohol use disorders (19%), and depressive disorders (19%). - Mental and substance use disorders were the top contributors to the disease burden for both sexes, accounting for 23% among males and 22% among females. - Mental and substance use disorders were the main contributors to the health burden during late childhood, adolescence, and adulthood among Indigenous Australians. | Not specified | National |
| 2 | AIHW, 2024 | Smoking (13.3%) and high body mass index (10.8%), together, accounted for 24.1% of the disparity in self-rated health between first Nations and non-Indigenous Australians aged 18-64 years population | Not Specified | 35% of the health gap between Indigenous and non-Indigenous Australians was explained by social determinants, including differences in education, employment, income, housing, and access to services. Additional factors such as food security and experiences of racism also contributed to this gap. | National |
| 3 | Australian Government & Productivity Commission, 2025 | - Among Indigenous Australians aged 65–74 years, coronary heart disease was a major cause of disease burden. - Among Indigenous Australians aged 65–74 years, chronic obstructive pulmonary disease was a major cause of disease burden. - Among Indigenous Australians aged 65–74 years, lung cancer was a major cause of disease burden. - Among Indigenous Australians aged 65–74 years, chronic kidney disease was a major cause of disease burden. - Among Indigenous Australians aged 65–74 years, type 2 diabetes was a major cause of disease burden. | Mental and substance use disorders accounted for 23% of the total disease burden and 42% of the non-fatal burden among Indigenous Australians. Within this category, anxiety disorders contributed 23%, alcohol use disorders 19%, and depression 19%. | - Social determinants explained 35% of the overall health gap between Indigenous and non-Indigenous Australians. - Employment status and hours worked accounted for 14.4% of the health gap between Indigenous and non-Indigenous Australians. - Equivalised household income accounted for 12.6% of the health gap between Indigenous and non-Indigenous Australians. - Level of school completion accounted for 8.9% of the health gap between Indigenous and non-Indigenous Australians. | National |
| 4 | AIHW, 2023 | - Chronic diseases were a major contributor to Indigenous mortality, especially CVD, Diabetes, Chronic respiratory disease (e.g., COPD), and kidney disease. - Between 2006 and 2015, deaths from cardiovascular disease declined among Indigenous Australians, while deaths related to diabetes increased, particularly among males. | Not Specified | - Indigenous Australians living in the most disadvantaged areas had a substantially higher death rate than those in less disadvantaged areas. - The life expectancy gap is partly attributed to social determinants such as education, employment, and income status | New South Wales, Queensland, Western Australia, Northern Territory. |
| 5 | AIHW, 2025 | Age-adjusted death rates from injury and poisoning were 1.6 times higher among First Nations people in Queensland compared with other non-Aboriginal people. The age-adjusted mortality rate from diabetes was 5.6 times higher among First Nations people in Queensland compared to non-indigenous. In addition, 2.8% of First Nations adults had been diagnosed with chronic kidney disease, while biomedical testing indicated that 3.4% were living with the condition. | - 31% of First Nations adults (18+) had high or very high psychological distress. - Between 2015 and 2019, the suicide rate among Aboriginal and Torres Strait Islander peoples was 26 per 100,000 population, with higher rates observed in males (39 per 100,000) than in females (13 per 100,000). | - Life expectancy for First Nations males was 74.6 years in the least disadvantaged areas vs. 69.5 in the most disadvantaged. - Life expectancy among First Nations females varied by socio-economic status, with women in the least disadvantaged areas living 77.0 years compared with 74.0 years in the most disadvantaged areas. However, even in the least disadvantaged areas, the life expectancy of First Nations people remained lower than that of non-Indigenous people in the most disadvantaged areas. Regional differences were also evident, as First Nations males in remote areas had a life expectancy of 67.3 years, which was notably lower than the 72.5–72.8 years observed among those living in major cities and regional locations. | New South Wales, Queensland, Western Australia, Northern Territory |
| 6 | AIHW, 2025 | - 67% of First Nations people reported at least one long-term condition. - 36% reported three or more conditions. - 240,000 years of healthy life lost, equivalent to 289 years of healthy life lost for every 1,000 aboriginal people due to illness, injury or early death in 2018. Top 5 contributors: Mental health & substance use: 23%; Injuries: 12%; cardiovascular diseases: 10%; Cancer: 9.9%; Musculoskeletal: 8% | Suicide accounted for 5.2% of all deaths among First Nations people, representing 7.1% of all male deaths and 3.0% of all female deaths. After adjusting for age, First Nations males experienced 48.5 suicide deaths per 100,000 population, which was 3.5 times the rate observed in First Nations females (13.8 per 100,000). Both First Nations males and females also had higher rates of suicide deaths than their non-Indigenous counterparts. Specifically, the age-adjusted rate for First Nations males was 2.9 times higher than that of non-Indigenous males, while the rate for First Nations females was 2.6 times higher than that of non-Indigenous females. | First Nations people in remote areas experience greater disease burden and shorter life expectancy than those in non-remote regions | New South Wales, Queensland, Western Australia, Northern Territory |
| 7 | Older Australians – AIHW 2021 | - Older Indigenous Australians experience an earlier onset of illness and a lower life expectancy compared to non-Indigenous Australians. - Among Indigenous Australians aged 50 years and older, 35% reported having diabetes or high sugar levels. - Approximately 19% (26,100 individuals) of Indigenous Australians aged 50 years and older rated their health as poor. - Indigenous Australians aged 65 years and older spend more than 50% of their remaining life with some form of disability. - Older Indigenous Australians lose an estimated 2.6 million disability-adjusted life years (DALYs) due to chronic physical diseases. | Not Specified | Not specified | New South Wales, Queensland, Western Australia, Northern Territory |
| 8 | AIHW, 2024 | Not specified | Not Specified | - 30% of First Nations people lived in the most disadvantaged areas vs. 8% of non-Indigenous - Only 2% lived in the most advantaged areas vs. 11% non-Indigenous - Disadvantaged areas are linked to health risks, psychosocial stress, and unhealthy behaviours | New South Wales, Queensland, Western Australia, Northern Territory |
| 9 | AIHW, 2024 | Not specified | Not Specified | Employment, income, and education explained 14%, 13%, and 8.9% of the health gap between Indigenous and non-Indigenous Australians, respectively. Among First Nations adults, being employed was associated with 1.5 times higher odds of reporting good health compared with being unemployed, and completing Year 12 was linked with 1.3 times higher odds of good health. Risk factors also contributed to disparities: smoking reduced the odds of good health by 30–40% for both Indigenous and non-Indigenous groups, while obesity accounted for 11% of the overall health gap. | National |
| 10 | AIHW, 2024 | - Not specified | - 31% of Indigenous adults experienced high/very high psychological distress. - 52% increase in mental health-related hospitalisations (2009–10 to 2018–19). - 4.3 times more likely to use Allied Psychological Services (2015–16). - 67% as likely to claim psychologist care via Medicare compared to non-Indigenous. | - Not specified | National |
