# Supplementary material for: Understanding the factors shaping Healthy Life Expectancy (HALE) in Indigenous Australians: A Scoping Review
Source: PLoS One. 2026 Nov 11;21(11):e0347718. doi: 10.1371/journal.pone.0347718 (PMC13754099; doi:10.1371/journal.pone.0347718)
Supplement: Supplementary file 4 [file pone.0347718.s004.docx]

**S4 Table. Summary of Government Reports and Scientific Papers on HALE, LE, and Quality of Life**

| SL # | Name of Author/Issuing Authority of the Report | Reported HALE/QoL/LE | HALE / QoL / LE Reported at Regional Level |
| --- | --- | --- | --- |
| 01 | Zhao et al., 2024 | HALE:  Male: Indigenous 43.3; non-Indigenous 69.9; Female: Indigenous 41.4; non-Indigenous 74.8  ULE:  Male: Indigenous 22.6; non- Indigenous 10.1; Female: Indigenous 27.4; non-Indigenous: 10.6 | Northern Territory |
| 02 | Zhao et al., 2022 | LE  Year: 1999  Male: Indigenous 56.6; non-Indigenous 77.4; Female: Indigenous 64.8; non-Indigenous 84.3  Year: 2018  Male: Indigenous 65.6; non-Indigenous 81.0; Female: Indigenous 69.7; non-Indigenous 85.1 | Northern Territory |
| 03 | ABS, 2023 | LE  Indigenous Male: 71.9; Female: 75.6 (National) | Male:  NSW: 73.8, QLD: 72.9, WA:68.9 NT: 65.6  Female  NSW: 77.9; QLD: 76.6; WA: 72.6; NT: 69.4 |
| 04 | AIHW, 2022 | HALE at 65 (2018):  Indigenous males: 9.1 years; non-Indigenous males: 14.6 years; Gap: 5.5 years  Indigenous females: 9.5 years; non-Indigenous females: 15.7 years; Gap: 6.2 years | Males  Indigenous – Major cities: 57.1, Inner/Outer regional: 56.1, Remote/Very remote: 53.1  Non-Indigenous – Major cities: 71.8, Inner/Outer regional: 70.3, Remote/Very remote: 71.1  Gap (Years – Major cities: 14.8, Inner/Outer regional: 14.2, Remote/Very remote: 18.1  Females  Indigenous – Major cities: 60.1, Inner/Outer regional: 58.9, Remote/Very remote: 55.7  Non-Indigenous – Major cities: 73.8, Inner/Outer regional: 72.6, Remote/Very remote: 70.8  Gap (Years) – Major cities: 13.7, Inner/Outer regional: 13.7, Remote/Very remote: 15.1  HALE at Age 65  Males  Indigenous – Major cities: 9.2, Inner/Outer regional: 9.6, Remote/Very remote: 7.0  Non-Indigenous – Major cities: 14.5, Inner/Outer regional: 14.9, Remote/Very remote: 13.8  Gap (Years) – Major cities: 5.2, Inner/Outer regional: 5.2, Remote/Very remote: 6.8  Females  Indigenous – Major cities: 9.9, Inner/Outer regional: 10.0, Remote/Very remote: 7.7  Non-Indigenous – Major cities: 15.6, Inner/Outer regional: 15.8, Remote/Very remote: 13.2  Gap (years) – Major cities: 5.7, Inner/Outer regional: 5.8, Remote/Very remote: 5.5 |
